# Supplementary figures and images for: Health Risk Assessment of PM2.5 and PM2.5-Bound Trace Elements in Thohoyandou, South Africa
Source: Int J Environ Res Public Health. 2021 Feb 2;18(3):1359. doi: 10.3390/ijerph18031359 (PMC7908426; doi:10.3390/ijerph18031359)

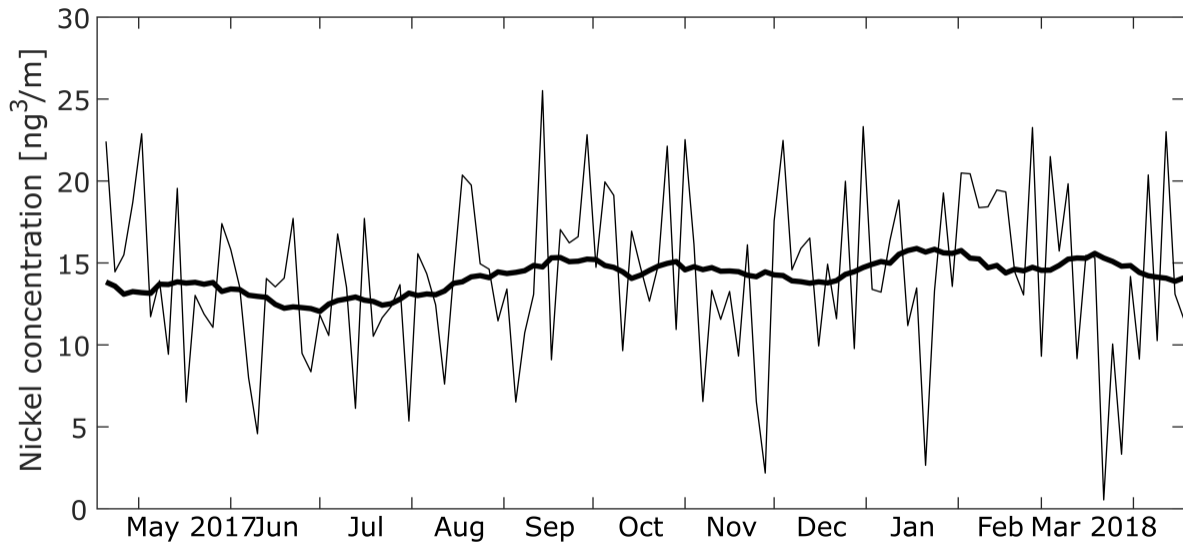

Supplement: Supplementary file 1 [file ijerph-18-01359-s001.pdf]
